# Utilizing rapid qualitative assessment and thematic analysis methods to identify and share promising case investigation and contact tracing practices with people in refugee, immigrant, and migrant communities during COVID-19

**DOI:** 10.3389/fpubh.2024.1359145

**Published:** 2024-07-03

**Authors:** Windy Fredkove, Erin Mann, Seja Abudiab, Diego De Acosta, Yesenia Garcia, Sarah J. Hoffman, Sayyeda Karim, Christine Thomas, Kimberly Kan-Yih Yu, Katherine Yun, Elizabeth Dawson-Hahn

**Affiliations:** ^1^Center for Global Health and Social Responsibility, University of Minnesota, Minneapolis, MN, United States; ^2^Department of Pediatrics, University of Washington, Seattle, WA, United States; ^3^National Resource Center for Refugees, Immigrants, and Migrants (NRC-RIM), University of Minnesota, Minneapolis, MN, United States; ^4^Seattle Children's Research Institute, Seattle, WA, United States; ^5^Population Health and Systems Cooperative, School of Nursing, University of Minnesota, Minneapolis, MN, United States; ^6^Division of Infectious Diseases, Department of Medicine, University of Minnesota, Minneapolis, MN, United States; ^7^Perelman School of Medicine, University of Pennsylvania, Philadelphia, PA, United States

**Keywords:** qualitative, needs assessment, immigrant, rapid analysis, thematic analysis, case investigation and contact tracing, migration, COVID-19

## Abstract

In the early months of the COVID-19 pandemic The National Resource Center for Refugees, Immigrants and Migrants (NRC-RIM) was established. NRC-RIM initially sought to rapidly identify promising case investigation and contact tracing (CICT) practices within refugee, immigrant, and migrant communities. Between September 2020 and April 2021, the team conducted 60 interviews with individuals from cross-sector organizations (i.e., public health, health systems, community experts/organizations) working with refugee, immigrant and migrant communities in health and public health capacities related to COVID-19. The overarching aim was to identify and amplify innovative promising and best practices for CICT with refugee, immigrant, and migrant communities, including an exploration of barriers and facilitators. We utilized layered methods to rapidly assess, summarize and disseminate promising practices while simultaneously completing four thematic analyses including: (1) public health organizations; (2) health system organizations; (3) community leaders and organizations; and (4) vaccine planning and access across the three sectors. The primary objective of this article is to describe the project design, applied methods, and team science approach we utilized. We found that rapid identification and dissemination of promising practices, and barriers and facilitators for CICT with refugee, immigrant and migrant communities was feasible during a public health emergency. This approach was essential for identifying and widely sharing culturally and linguistically concordant public health practices.

## Introduction

In the early stages of the COVID-19 pandemic, case investigation and contact tracing (CICT) was the primary public health intervention. At that time there were few well described best practices for conducting CICT with people in refugee, immigrant, and migrant (RIM) communities across the United States. Specifically, program descriptions of comprehensive, culturally and linguistically matched CICT practices were lacking to support people in RIM populations who were already experiencing disproportionate risk for COVID-19 (1) due to myriad systemic factors, including access to information in languages other than English (2, 3) and frequently professions as essential or front-line workers (4). To address this gap, the National Resource Center for Refugees, Immigrants, and Migrants (NRC-RIM) was established at the University of Minnesota in October of 2020 with multiple collaborators and external faculty funded by the US Centers for Disease Control and Prevention (CDC) and the International Organization for Migration (IOM) (5). NRC-RIM is a resource center for public health and community-based organizations serving refugee, immigrant and migrant communities (5).

To understand how CICT was working with RIM communities the team recognized the need to quickly learn about and share the approaches public health, health systems and community-based organizations were utilizing with CICT. Accordingly, NRC-RIM created a multidisciplinary Qualitative Collaborative team focused on collecting qualitative interview data from people across the United States who were engaging in CICT with RIM communities that would simultaneously inform a rapid assessment *and* dissemination of potential promising practices [i.e., *strategies, approaches, or programs that have anecdotally shown to have a positive impact in local settings* (6)].

Quickly responding to emergent public health crises “is vital to reducing their escalation, spread, and impact on population health” [(7), p. 1]. Additionally, during complex public health emergencies, rapid data collection and analysis approaches can be effectively utilized to share information (8, 9). Since there was sparse literature when our team began considering the need for and benefits of a combined methodological approach (i.e., rapid and more traditional qualitative analysis), the Qualitative Collaborative selected and adapted a rapid qualitative approach (10, 11) paired with a thematic analysis (12). In this paper, we describe this layered analytic approach as a rapid assessment and dissemination (RAD) of promising practices with a concurrent thematic analysis (TA). We systematically documented our methods to (a) bolster assessment and analytic consistency (i.e., a shared process structure across the team), (b) enhance transparency and rigor, (c) describe methodological decisions and (d) highlight the interdisciplinary team approach (13, 14). We are hopeful this description of methods will support others' planning for and responding to urgent public health issues.

## Methods

### Project aims

The overarching objective of this qualitative needs assessment project was to reduce healthcare disparities and promote health of RIM communities during the COVID-19 pandemic. The team focused on understanding the perspectives and strategies of organizations working with RIM communities across three sectors: public health, health systems and community-based organizations. The project had two aims: (1) to identify best and promising practices for comprehensive CICT among RIM communities; and (2) to identify facilitators and barriers related to CICT with RIM. We describe the step-by-step procedures of this layered qualitative methods approach using this project as an applied example.

### Design

Traditionally, case investigation and contact tracing has focused primarily on those two activities; however, our approach intentionally utilized a broader, more inclusive definition of what we describe as *comprehensive CICT*. Our team defined comprehensive CICT as “the continuum of engagement with public health organizations to support people who were infected with or exposed to COVID-19, including culturally responsive strategies such as health education and communication, testing, case investigation, contact tracing, quarantine and isolation, health monitoring and resource provision” [(15), p. 2].

The team sought to conduct semi-structured interviews with organizations that were conducting components of comprehensive CICT across public health, health systems and community organizations. The same qualitative project design was used for each set of sector-specific interviews, for a total of 60 interviews.

### Participants

We utilized a purposive sampling approach with geographic stratification across the ten United States Department of Health and Human Services regions (16) to capture perspectives from across the US, including at least one interviewee from all 10 regions for the set of public health professional interviews (15), and all but one region represented in each of the other two sets of interviews (2, 17). The team engaged with interviewees from organizations in varying geographic settings (e.g., rural, urban, suburban, near the US-Mexico border), serving different communities (e.g., specific immigrant communities, recently resettled refugees, migrant farm workers), and with a broad representation of languages spoken. Eligibility was based on being actively involved with a public health, health system, or community organization that was providing some component of the comprehensive COVID-19-related CICT services with RIM communities.

Recruitment of potential interviewees and scheduled interviews were maintained within a spreadsheet and discussed at weekly team meetings. Potential interviewees were identified through a network of public health practitioners and health care providers known by the project team, through the Society of Refugee Health Providers listserv, American Academy of Pediatrics Council on Immigrant Child and Family Health listserv, the Association of Refugee Health Coordinators, the NRC-RIM Community Leadership Board, and later through recommendation of already participating interviewees. Participants from the community organization cohort received compensation in the form of a gift card.

### Ethics approval

This project was deemed non-human subjects research by the University of Minnesota and exempt by the University of Washington.

### Interview guides

The semi-structured interview guides, accessible within each individual study publication (2, 15, 17) were intentionally comprehensive, including questions spanning a continuum of CICT activities and processes, and in alignment with the team's definition of *comprehensive CICT* (15). We asked interviewees to provide their perspective as a professional working within an organization. The initial interview guide, and subsequent iterations of the guide where adjustments were made to tailor questions for each interview cohort, were developed collaboratively. The interview guides included questions about vaccination to begin exploring how organizations were thinking about this anticipated next phase of the public health response (18).

### Data collections

Recruitment and interviews were initiated in September 2020 beginning with (1) public health organizations, followed by (2) health systems and then (3) community experts/organizations. Notably, due to the rapid unfolding of the pandemic and need for swift action and information sharing, some core NRC-RIM team members began conducting interviews as soon as institutional review board approvals were received. The founding team of NRC-RIM anticipated the establishment in October 2020 and initiated the interviews as a way to inform the structure, focus and early activities of the center. Recruitment and interviews were conducted throughout the project until all interviews were completed in April 2021.

Before initiating the interview, interviewers provided a summary of the project, described the data collection and storage process, the use of data after transcription, and asked permission to conduct and audio record the interview. Each participant provided demographic information through a REDCap questionnaire (19, 20). All interviews were conducted in English through Zoom. A total of 60 interviews were conducted, including local and state public health organizations (*n* = 21), health service providers across specialties and settings (*n* = 20), and community experts and community-based organizations (*n* = 19) [see (2, 15, 17) for demographic data]. Data was primarily collected through semi-structured interviews; however, interviewers often took field notes during the interviews, and interviewees would occasionally share relevant documents or resources.

### Data management

The interviews were audio recorded, professionally transcribed, and uploaded into a secure, cloud-based database. During and immediately after the interview, each interviewer took detailed notes and summarized the interview data using a structured summary template. Summary documents were then uploaded to cloud-secured team folders. A de-identified copy of each transcript was also uploaded into the qualitative software, Dedoose version 9.0.107 (21), which was used for data management and analysis. Specific team members were given access to the cloud-secured folders and/or the qualitative software based on the need to perform operational or analytic tasks.

### Timeline

**Figure 1:**
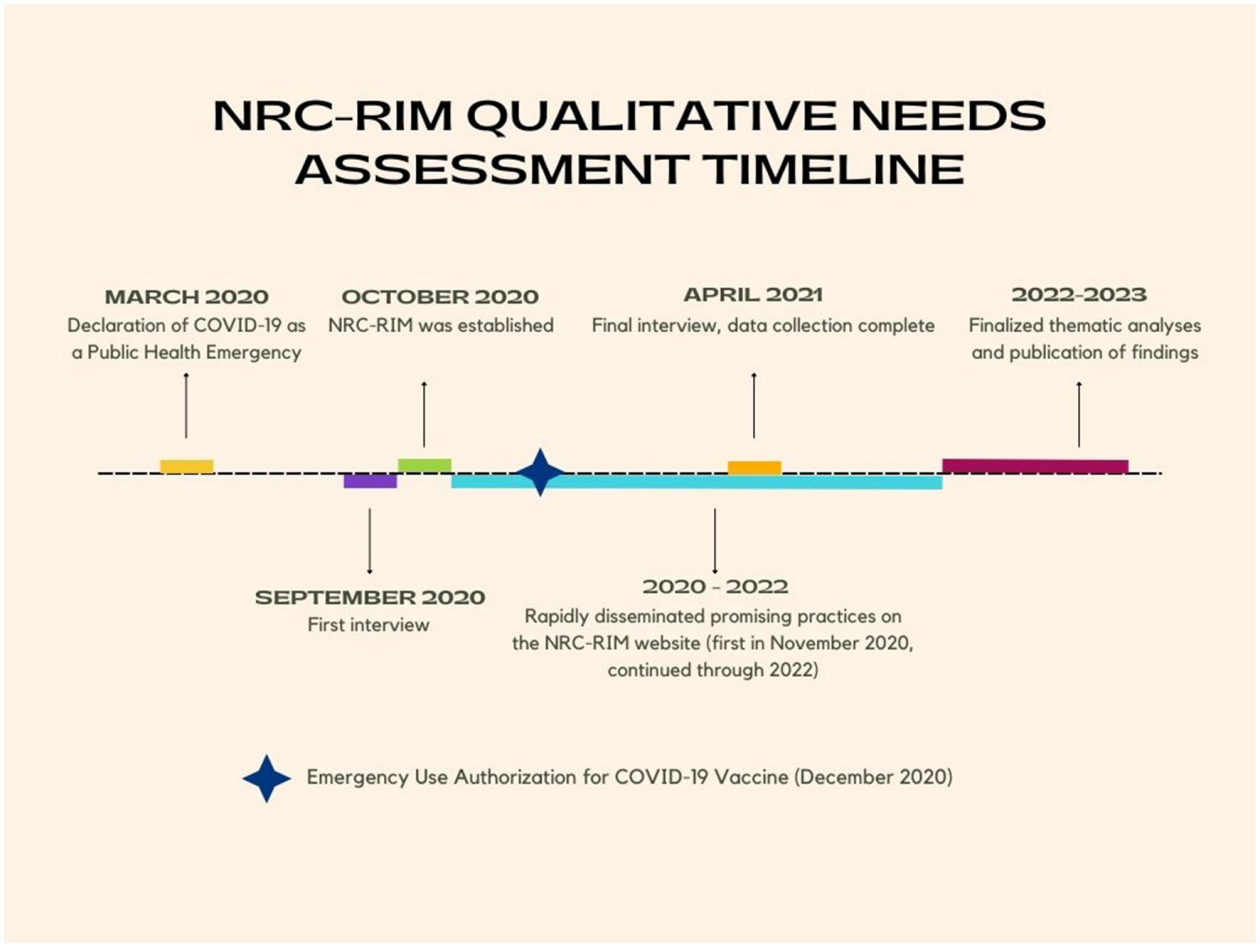
NCR-RIM qualitative needs assessment timeline.

Data collection (September 2020 through April 2021) occurred concurrently with the rapid assessment, summarization and dissemination of promising practices and the thematic analysis (12). Figure 1 provides an abbreviated visual timeline.

### Data analysis and dissemination, parallel approaches

**Figure 2:**
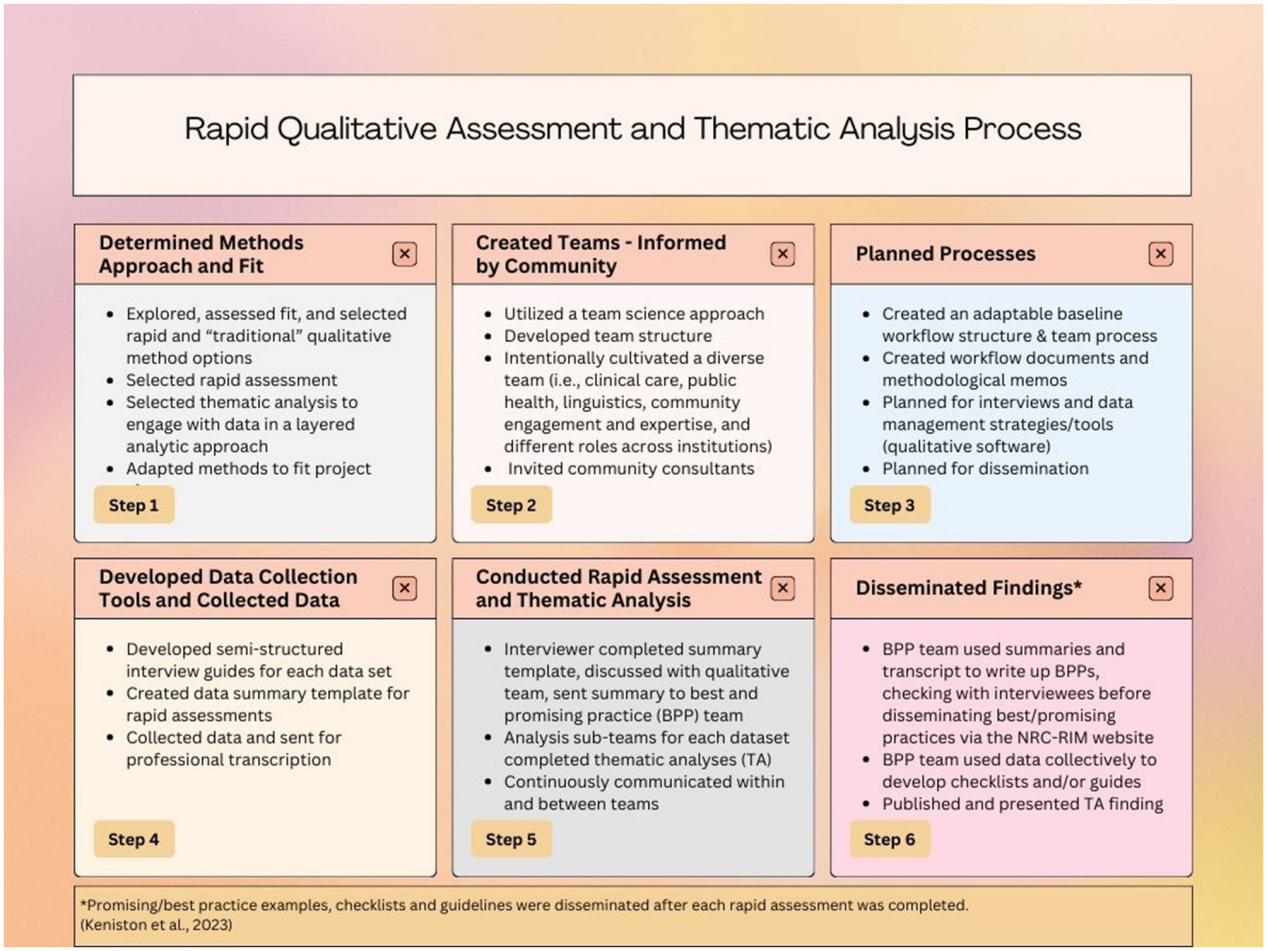
Rapid qualitative assessment and thematic analysis process (22).

We paired a rapid assessment and dissemination (RAD) approach to exploring and sharing promising RIM-specific CICT practices with a parallel thematic analysis (TA). The RAD-TA approach allowed our team to simultaneously collect interview data, expeditiously assess, summarize and share promising practices, while using thematic analysis methods to expand and augment what was known about CICT with immigrant communities across sectors. The parallel data analysis and dissemination approach necessitated two sub-teams within the Qualitative Collaborate. Figure 2 provides summarized process steps presented linearly for clarity; however, some steps occurred simultaneously.

### Qualitative collaborative development and processes

#### Team science

The Qualitative Collaborative at NRC-RIM employed a team science approach (13, 14, 23), and is made up of people with expertise in including clinical care (medicine and nursing), public health, linguistics, community engagement, leadership, and education; with several roles including academic faculty, fellows, graduate and undergraduate students, project managers and consultants, and community experts. Several team members had extensive experience focused on migration in clinical or public health spaces, identified as multilingual, and/or were from RIM communities.

The team science approach also promoted a collaborative learning environment wherein the more research- and evaluation-experienced team members provided guidance and training for members newer to qualitative methods. Those with less research and evaluation expertise shared expertise from their field and lived experience. Additionally, the team developed a communication process and written guides that supported a collective understanding of methodological decisions, resources and method processes. Importantly, the team leads were intentional about co-creating a welcoming virtual environment, including providing an agenda with opportunity for adaptation as needed, offering time and space to discuss current issues related to the pandemic and/or any personal/professional celebrations, as well as recognizing and reflecting on the team's collective progress.

#### Qualitative collaborative

The Qualitative Collaborative, situated within NRC-RIM, was made up of two subteams to facilitate the parallel data analysis and dissemination approach: (1) the *qualitative team*, with 10–14 members (varying number depending on what point in the project), conducted recruitment, interviewed, summarized interviews and conducted the subsequent thematic analyses; and (2) the *best and promising practices (BPP) team*, comprised of 6–8 of team members, focused on writing up best and promising practices for dissemination on the NRC-RIM webpage. Rapid dissemination occurred through a promising practice write-up or integrating learnings into guidelines and checklists. Thematic analysis findings were disseminated through peer-reviewed manuscripts and presentations.

The team science approach, and specifically within and cross-team communication, was essential to the success of this project. The Qualitative Collaborative team lead and subteam lead attended broader NRC-RIM team meetings and shared information back with the qualitative and BPP teams. Occasionally, members of one subteam would join the other to enhance communication about the interviews, summaries, or processes. Once thematic analyses began, the qualitative team was further split into dataset-specific subteams, while continuing to meet weekly as a whole team.

#### Community leadership board and community consultants

The NRC-RIM Community Leadership Board (CLB) provided consultation and guidance to the Qualitative Collaborative. The CLB was composed of community members from several refugee, immigrant, and migrant communities in the US who had a history of work with public health or health systems. The CLB provided advice for recruitment of interviewees and members of the CLB were a part of the analysis teams discussing assignment of themes, framing of findings and co-authoring products – manuscripts, abstracts, and presentations. Each thematic analysis team included one consultant from the CLB – the public health professional analysis team included a public health practitioner, the health systems team included a health provider, the vaccine analysis team included a community leader with vaccine outreach experience, and the community experts/organizations analysis team included two community expert interview participants. The time and expertise of the CLB members and community consultants was valued with a stipend.

### Rapid assessment, summary and dissemination of best and promising practices

Following each interview, the interviewer reviewed their notes and the transcript, then wrote a summary utilizing a template. The interview summary template was adapted from Hamilton's rapid analysis methods (10, 11); however, we describe it as a *rapid assessment* because we did not conduct a formal analysis at this phase. The summaries were stored in the secure storage site.

Once each summary was complete, the Project Coordinator contacted the best and promising practices (BPP) team. The BPP team met weekly to discuss the interview summaries, identify potential best practices (supported by existing evidence in the literature) or promising practices (successful approach not yet supported by evidence). The team then prioritized the order of writing up the BPP's for dissemination. The lead author for each BPP reviewed the summary and the interview transcript in order to write the promising practices. The promising practices were 800–1500 words in length and provided a general overview of a topic area. These included specific details and additional resources (when available) sourced from interviews to support other organizations interested in replicating the practice (24). One team member led writing the BPP then it was reviewed and edited by 1–2 other team members. Once a draft was complete, the lead author contacted the interviewee and/or organization to review the proposed BPP write-up. The BPP team asked the interviewee to add any feedback, ensure accuracy of the information included, and requested permission to disseminate the BPP on the NRC-RIM website. The promising practices were added to the NRC-RIM website (6) and informed the creation of new guides and checklists for public health practitioners and community-based organizations. The iterative process described here ensured the credibility and trustworthiness of the rapid assessment and translation of the data into disseminatable products to support the evolving pandemic landscape.

### Thematic analysis

We conducted multiple thematic analyses to further describe and expand our understanding of the processes underlying interviewees' CICT response experiences and promising practices within RIM communities, including related facilitators and barriers (12). Our multidisciplinary team completed four related, but separate thematic analyses (2, 15, 17, 18). The clear and flexibly structured guidance provided by Braun and Clarke's (12) thematic analysis method was an ideal fit for the needs assessment focus of this NRC-RIM project and a team science approach.

**Figure 3:**
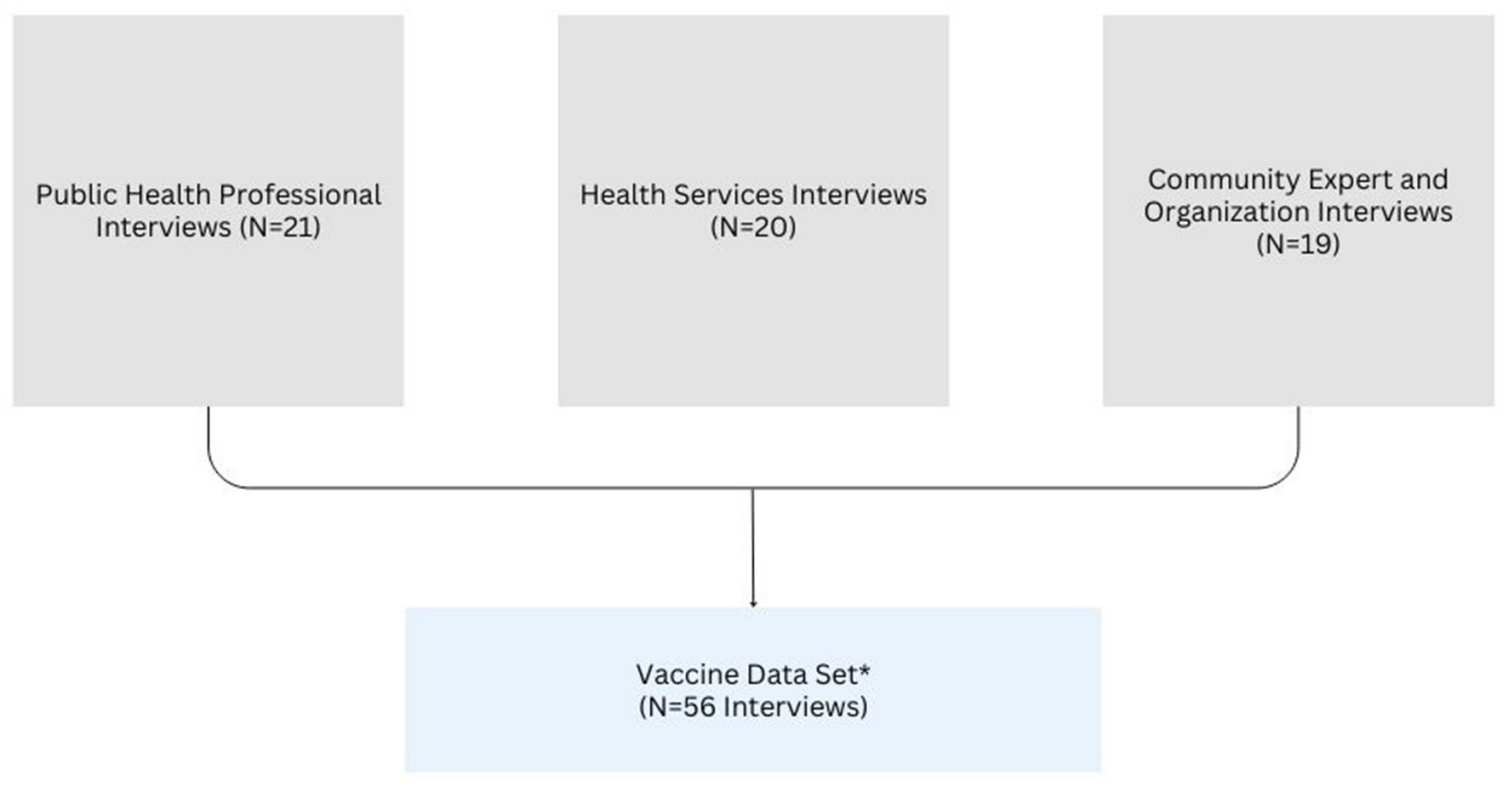
Data set structure. ^*^Vaccine data set only included data from interviews that contained vaccine related data (i.e., four transcripts from the three data sets were not included in the vaccine data set).

**Figure 4:**
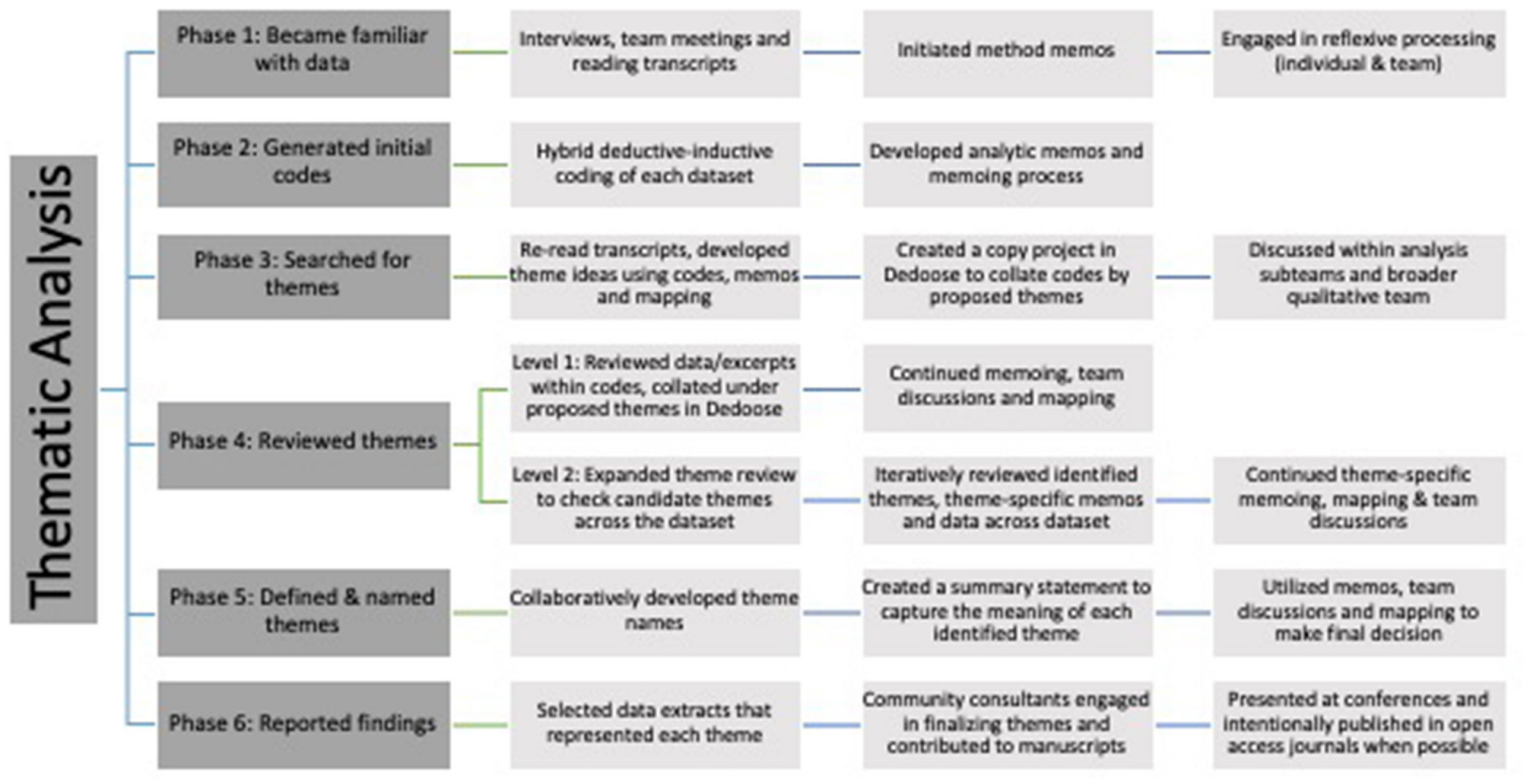
Thematic analysis process (12).

One thematic analysis was completed with data for each of the three interview groups: public health, health systems and community experts/organizations, while the fourth focused on vaccination data sourced from across all three interview groups (Figure 3). Analysis for the public health practitioner dataset (the earliest set of interviews) was initiated first, followed by analysis of the health systems providers and community expert/organization data as interview transcripts became available for analysis. Lastly, the team conducted the cross-sector analysis of vaccine-related data across all three datasets. In the following sections, and in Figure 4, we summarize the activities our team engaged in through each of Braun and Clarke's (12).

#### Phase 1: familiarizing yourself with data

For each thematic analysis two or three team members followed the phases identified by Braun and Clarke (12). This began by becoming familiar with and immersed in the data, including “reading the data in an *active* way” to facilitate the search for meaning and patterns. Each team member engaged in the initial familiarization phase by (1) conducting the interviews and rapidly summarizing interview content, (2) engaging in post-interview team discussions, and/or (3) by “reading and re-reading” transcripts [(12), p. 87]. Accordingly, all team members engaged in conducting data analysis read every transcript in the dataset they were working with (12). An overarching team memo on methodological process and decision making was also initiated during this phase. Throughout the thematic analysis process, our team members considered and engaged in reflexive processing around method approaches and decisions, as well as our unique perspectives, contexts, experiences, and positionalities (25).

#### Phase 2: generating initial codes

The next step was identifying patterns, which was facilitated by creating a flexible CODEBOOK for each set of data. Though creating a codebook is not typical of Braun and Clarke's approach to TA (26, 27), in our assessment of the project needs, working with multiple datasets, and engaging in a tailored team science process, the codebook approach was an appropriate and useful adaptation. Initial coding in the public health professional dataset analysis process was completed without an *a priori* codebook, but was deductive in that it was reflective of the interview transcripts mirroring the semi-structured interview guide. The public health professional interview data served as a foundational codebook for the subsequent analyses because the semi-structured interview guides for each dataset were based on the initial public health professional interview questions (with additional relevant adaptations based on the cohort of interviewees).

Teams only used codes from the original codebook if, and when aligned with the data in their specific dataset. The coding teams each conducted inductive coding as well. The coding approach was predominantly semantic [i.e., explicit rather than extending to a more latent, interpretive level of analysis (12)]. The fourth analysis was unique in that it focused on a compilation of cross-sector vaccine-related data. The team followed Braun and Clarke's (12) recommended steps to “coding” without a codebook for the fourth analysis rather than utilizing the original coding scheme.

For each of the first three analyses (i.e., public health professionals, health service providers, community experts/organizations), coding was completed in Dedoose (version 9.0.107) (21). The process began with the first five transcripts being independently coded by two coders. This approach informed the iteratively developed codebook, discussions around codes and definitions, and the initiation analytic memos. Subsequently, one of the five transcripts coded by two independent coders was selected to be reviewed in full (i.e., comparing and discussing each coded excerpt, moving excerpt by excerpt through the entire transcript) so as to establish a consistency in the approach, as well as an understanding of the data and how each team member was interpreting and applying the codes (i.e., embracing differences of interpretation and application using a constructivist and reflexive approach). Notations related to these team discussions were made using a combination of notes within the codebook structure in an Excel spreadsheet. Codebook versions were maintained for process and content review as needed. Additionally, teams utilized Dedoose annotations and memos to track places for discussion and interpretation of data.

Each of the original data analysis subteams (i.e., public health, health systems, community expert/organization) created and took an inter-rater reliability (IRR) test utilizing the Dedoose Training Center feature (28). To complete the IRR test, we consulted the IRR module within Dedoose (28), as well as O'Conner and Joffee's (29) perspective and guidelines. Ultimately, IRR in our project was aimed at supporting team members new to data analysis, which was aligned with our team science approach. This also supported consistency in coding broadly, while honoring the unique perspectives each analyst contributed to the analysis. Once the IRR test was completed with a satisfactory score (focused on the confidence of the team and coder), the team reviewed the results and one coder proceeded to independently code the remaining transcripts for that dataset. Critical to this process is that there were always two team members available (i.e., one of the team leads and a cross-sector coding team lead) to guide each of the data analysis subteams with coding and logistical questions concerning Dedoose. Additionally, the coding cohorts met every week to discuss coding or process any questions. During these meetings the coder and the other team members used the annotation and memo features in Dedoose to guide the discussion and make ongoing analytic decisions. These memos became critical in the next phases of theme development.

The analysis of the vaccine-related data was unique in that although the interviews were focused on CICT efforts and practices, questions were added in anticipation of the roll out of vaccines. Accordingly, the three sector-based coding teams created a general parent code to capture vaccine data. As data collection and coding progressed, the team decided to do a cross-sector analysis of vaccine-related data. Subsequently, as the interviews and analyses were occurring concurrently, the teams were more attuned to specifically coding vaccine-related data into a broad “vaccine” code, with a few vaccine-related subcodes/child codes when clear patterns were observed. The two team members analyzing the subset of vaccine related data were already familiar with the data through conducting interviews and summaries, or having read transcripts and coded in support of, one or more of the sector-specific datasets. The vaccine data analysis team systematically reviewed the vaccine-relevant data excerpts pulled from each of the datasets through using a spreadsheet, allowing the team to code excerpts and begin collating the data into inductively developed codes in preparation for the next phase.

#### Phase 3: searching for themes

In this phase our team engaged in the process of sorting codes into themes, “collating all the relevant coded data extracts within the identified themes” and continued “thinking about the relationship between codes, between themes, and between different levels of themes” [(12), p. 89]. Each teams' memos and excerpt-linked annotations within Dedoose aided in this process.

An independent review of codes and potential themes was completed wherein each analyst took time to look over every code and create an individual memo attempting to collate the codes into cohesive themes. After completing this step independently, team members met to compile, discuss and make decisions about theme selection from the pool of potential themes they developed. Each analysis team met to discuss individual impressions of the codes and potential overarching themes. The team completed this step by combining individual memos and discussion notes onto one collaborative document. Some of the analysis teams utilized a virtual whiteboard tool to sort theme ideas and begin developing thematic maps, while other analysis teams primarily used the collaborative group document to sort through theme ideas.

During this phase, an original version of each coded transcript was maintained. A copy Dedoose project was created to allow for more freedom in the iterative code and excerpt collation process to aid theme development during this phase. One team member collated the codes in Dedoose based on the team's discussions and decisions.

#### Phase 4 (levels 1 and 2): reviewing themes

The process of considering potential themes continued with Phase 4, Level 1 (code-informed themes) and eventually to Phase 4, Level 2 (analyzing themes across the dataset). During Level 1, each data analysis team reviewed themes by comparing data from excerpts within the codes collated under each proposed theme. Using the duplicate Dedoose copy of each dataset, organized by *candidate* themes, analysis teams (1) reviewed all excerpts under each candidate theme, (2) wrote thematic memos, and (3) created theme review memos for other analysis team member(s) to review and respond to during asynchronous analysis and/or during team meetings. Team members also memoed (outside of Dedoose in a Word document) about potential subthemes or other relevant patterns.

Next, each data analysis team progressed to the second level of Phase 4 wherein the focus shifted to analyzing theme relevance and integration across the entire dataset, as opposed to the code-centered excerpt-to-excerpt comparisons completed during Level 1. Each analytic team continued this iterative process of discussing, identifying and reviewing candidate themes by moving back and forth between (a) the preliminary candidate themes organized in Dedoose, (b) the theme-linked memos and annotations within Dedoose, and (c) the shared theme-specific memo documents (shared Word documents). During this phase data analysis teams continued developing memos of all types (e.g., methods, analytic), and met weekly or every other week, frequently discussing and updating themes with the larger qualitative team. Additionally, the teams engaged in thematic mapping (i.e., creation of visual depictions of themes and thematic relationships), sometimes diagramming manually on paper, within a shared Word document, or with an interactive virtual map (e.g., Jam Board). Examples of final figures derived from the thematic mapping process can be found within each thematic analysis manuscript (2, 15, 17, 18).

#### Phase 5: defining and naming themes

The thematic definition and naming process of Phase 5 focused on refining the details of each theme, as well as “the overall story the analysis tells, generating clear definitions and names for each theme” [(12), p. 87]. Each data analysis team collaboratively developed theme names based on the codes and data subsumed under each theme, any thematic memos, the overall meaning of the theme, sometimes selecting direct quotes (i.e., *in vivo*) as theme names. Each analysis team created a summary statement that captured the key meaning for each identified theme, read through memos, reflected on the theme names and summary statements, and developed thematic maps to further refine theme names and definitions. Themes were then presented to the qualitative team in advance of the next meeting, so the team had time to review and reflect before discussing responses during subsequent large group qualitative team meetings. If any major questions or suggested changes were noted, the data analysis teams returned to the data until the team reached a shared understanding and agreement around theme name and meaning.

#### Phase 6: producing the report

Braun and Clarke describe Phase 6 as a “final opportunity for analysis,” and they encourage analysts to select “vivid, compelling extract examples” that ultimately relate the analysis back to the original inquiry question and the literature [(12), p. 87]. Each data analysis team used an iterative approach of selecting data extracts that would highlight and represent each theme, continuously considering how the themes and excerpts related to both the dataset and the parent project. One to two community consultants joined each analytic team to finalize themes, contribute to manuscripts and presentations of the final results. The team, in partnership with community consultants, collaborated to incorporate community expertise and perspective into the data analysis, interpretation and communication of the results, with careful attention to our approach to language and communication (30, 31).

## Discussion

This multidisciplinary qualitative project utilized rapid assessment and dissemination of promising practices and thematic analysis (i.e., RAD-TA approach) to elevate and extend our understanding of comprehensive CICT and vaccination strategies in RIM communities. This approach integrated (a) rapid qualitative data collection and assessment strategies that were adapted for this public health project's specific context and objectives, (b) a prominent research methodology (thematic analysis) to guide/an analysis of the data collected, and (c) a collaborative approach leveraging the multi-disciplinary expertise of the study team and community consultants. This layered approach led to the rapid dissemination of promising practices enabled sharing of approaches, guidance and resources for public health, health systems and communities engaged in CICT while the pandemic was unfolding. Simultaneously, the thematic analyses contextualized the best and promising practices in addressing barriers and incorporating facilitators to comprehensive CICT and vaccination.

There has been an expansion and advancement of rapid qualitative methods in the context of urgent public health situations (8, 9, 32). In recent years, however, much of the rapid qualitative analysis literature has centered on either rapid analysis as a singular method or rapid approaches in comparison to more “traditional” qualitative methods, leaving a gap in understanding how such rapid and traditional methods might be used together (33), as we attempted to do with our analysis. Utilizing a rapid qualitative analysis approach has the potential to address some of the challenges (e.g., time and resource limitations) that typically accompany more traditional qualitative methods (33, 34). Similar to other projects utilizing rapid qualitative methods (8), the NRC-RIM Qualitative Collaborative engaged in a rapid qualitative assessment with the intention of providing guidance and strategy for public health, healthcare and community-based organizations, e.g., promising practices (24) and guidelines and checklists (6). Pairing this style of rapid assessment with the thematic analysis was intended to begin to more broadly understand and contextualize promising practices for CICT with RIM communities, while recognizing the value of hyper-local and culturally specific practices. Our attempt to layer methods in this way is unique but aligned with others similarly attempting to integrate both types of analysis (33).

The uniqueness of our approach includes the ways the center's development, and the evolving activities, were continuously considered and integrated into NRC-RIM as the center continued to expand the community-engaged, multi-disciplinary, team science approach. For example, perspectives from interviews helped inform cross-sector emphasis of other NRC-RIM work, as well as ongoing and reciprocal conversations with the CLB about learnings from interviews and from the CLB back to the interviews (e.g., informing questions, processes). Multiple NRC-RIM teams were also represented when attending different components of the center's meetings wherein there was often a rich cross-pollination of ideas. Additionally, ongoing conversations were facilitated with each organization that had a BPP written about and highlighted through NRC-RIM, which allowed us to learn more about what the practices they were implementing.

### Limitations

While there are many benefits to the layered methods of qualitative data analysis, our experience with these methods illuminated some limitations of the RAD-TA approach. Our team carefully considered and weighed the strengths and limitations of rapid assessment methods generally and the specifics related to the RAD-TA approach. Though rapid data collection and analysis during an emergency response offers a path to explore and share current data, the benefits are accompanied by the need to grapple with a range of challenges (e.g, time constraints for practitioners/experts during an urgent response, need to adapt interview guides as the pandemic unfolded) that require pragmatic decision making. Layering a thematic analysis of the same data that was rapidly assessed adds depth and nuance, yet increases the complexity to the analytic process.

Through our RAD-TA method we attempted to expand our understanding of the data beyond the rapid assessment; however, we made the pragmatic choice not to include documents shared by interviewees nor the field notes or rapid summaries developed by our qualitative interviewers into the thematic analysis. Thus, we describe our method as layered rather than an integrated analysis. In future applications of this method and process, when pragmatically feasible, integrating the rapid assessment data (i.e., summaries, documents) into the thematic analysis has the potential to yield even more depth and analytic cohesiveness.

Another potential limitation is that the rapid assessment results (summaries and promising practice “write-ups”) may face selection bias due to the professional preparation and academic setting in which most of the qualitative team works. However, we likely mitigated some level of potential bias in the selection of promising practices and thematic analyses by creating a diverse team and being intentional about a community-engaged approach when possible. Finally, although measurement and evaluation are essential to demonstrate evidence-based (“best”) practices, anecdotal and community-defined successes are indispensable measures to consider as well. Moreover, evaluation and measurement of innovative and rapidly adapted promising practices are an key to understanding what programs deem as successful. Thus, the promising practices disseminated by NRC-RIM, may not yet be identified as “evidence-based,” have the potential to be key components of a community-centered public health emergency response (35, 36), ultimately promoting innovative, community-driven practices (often already feasible and acceptable within communities), with the potential for sustainability, and an opportunity for future evaluation (37).

### Innovative approach to community informed rapid dissemination

Though the foundation of conducting this project by way of community-engaged team science and utilizing a RAD-TA approach to data analysis are significant and essential to the project as a whole, the rapid dissemination model developed through NRC-RIM is particularly valuable in that the original intention of the project quickly came to fruition in service of communities and practitioners. Our team proactively planned for swift and accessible dissemination products during the design phase of the project, and continuously consulted RIM communities and RIM serving organizations throughout each stage of dissemination. We did this by developing dissemination products specifically for community and practitioner audiences distributed on the NRC-RIM website, including a review step with interviewees before disseminating promising practices, and including community consultants in thematic analysis, presentations, and manuscripts. We selected open access journals so that those who are generating the practices and knowledge being shared through this project will also have access to the reports.

### Reflections on utilizing a team science model in public health quality improvement

While operationalizing a project through a team science model is advantageous in many ways, it requires careful planning, consideration, and continuous stewardship. By building a multidisciplinary, diversely trained, and experienced team, we were able to bolster the project development, processes, methods, and dissemination, in addition to the depth that was achieved by way of the multiple perspectives contributed by each team member. The multidisciplinary and multicultural team provided the opportunity to approach development of the project, data collection, analysis, and dissemination through a variety of lenses. This led to a broader perspective and greater depth of exploration and understanding of data shared by participants, as well as how the data was analyzed and disseminated. We were able to lean on and learn from the strengths of team members given their expertise and experience. The inclusion of community consultants also brought together multiple perspectives and assets, while creating a safe and welcoming professional community, which is especially critical when working virtually. The flexibility of team members and the team structure allowed for rapid adaptations as needed, a cohesive approach to decision making, collective and individual team member skill building, and opportunities for growth and innovation that would not have been possible without utilizing a team science approach. Upon reflection, our team experience closely aligns with the 10 characteristics of a “good team” described in the work of Nancarrow et al. (14).

### Implications and recommendations

Importantly, these methods have a broader scope than the example project presented, with impact beyond the context of COVID-19. For teams primarily interested in rapid qualitative methods we suggest consulting Hamilton's work (10, 11) and the framework provided by Keniston et al. (22) (which we used as a base conceptualization for describing our layered analytic approach in Figure 2), as well as integrating rapid qualitative analysis steps with the consolidated criteria for reporting qualitative research (COREQ) (38). For other teams wanting to engage in a layered approach to data collection and analysis using both rapid and traditional qualitative data analysis, Suchman et al. (33) provides a comprehensive list of recommendations, including the integration of human centered design processes, especially in the context of complex global health studies. We also suggest proactive considerations of budget and funding to promote sustainability of projects current and potential future activities. This step is critical for organizations and community experts to be included in public health in a way that values the time expertise, and resources utilized.

## Conclusion

There is a need for transparent and detailed accounts of how qualitative needs assessments can be conducted during a public health emergency response with a multidisciplinary team. This qualitative needs assessment and promising practice dissemination project provided valuable resources for public health and community-based organizations serving refugee, immigrant and migrant communities during and beyond the COVID-19 pandemic. More broadly, these efforts were done with a focus on community health equity, as well as the intention of, and attention to, democratizing the dissemination of findings.

## Data availability statement

The raw data supporting the conclusions of this article will be made available by the authors, without undue reservation.

## Ethics statement

This project was deemed non-human subjects research by the University of Minnesota and exempt by the University of Washington.

## Author contributions

WF: Writing – review & editing, Writing – original draft, Conceptualization. EM: Writing – review & editing, Writing – original draft, Conceptualization. SA: Writing – review & editing. DD: Writing – review & editing. YG: Writing – review & editing. SH: Writing – review & editing. SK: Writing – review & editing. CT: Writing – review & editing. KK-YY: Writing – review & editing. KY: Writing – review & editing. ED-H: Writing – review & editing, Writing – original draft, Conceptualization.
